# Bone Mineral Density Measurements and Fracture Prediction in the Very Elderly

**DOI:** 10.7759/cureus.97458

**Published:** 2025-11-21

**Authors:** Jeff Borenstein, Jelena Maletkovic, Jarod DuVall, Maryam Sharifi

**Affiliations:** 1 Division of General Internal Medicine and Health Services Research, University of California Los Angeles David Geffen School of Medicine, Los Angeles, USA; 2 Division of Endocrinology, Diabetes, and Metabolism, University of California Los Angeles David Geffen School of Medicine, Los Angeles, USA

**Keywords:** bone mineral density, dual-energy x-ray absorptiometry (dexa), fragility fracture, osteoporosis treatment, very elderly

## Abstract

Bone density decreases with age, increasing the risk of fragility fractures. Dual-energy X-ray absorptiometry (DEXA) scanning may be used to measure bone density and estimate this risk. Degenerative changes in the spine may decrease the accuracy of DEXA results. We present the clinical course of a 92-year-old woman with a diagnosis of osteopenia of the left femoral neck and a risk for fragility fracture that was previously below the threshold for treatment, who underwent routine follow-up bone density screening. The DEXA scan showed an increase in bone density at the lumbar spine. Within a week of this study, she experienced severe back pain rated eight to nine out of 10 without a precipitating event. The following day, a lumbar X-ray showed disk space narrowing at multiple levels, spondylolisthesis at L4/5, diffuse spondylosis, and a new compression fracture at T12. This case illustrates the challenges of preventing and treating low bone density in the very elderly.

## Introduction

Loss of bone density typically begins between ages 50 and 60 years in most people, is usually progressive, and represents one factor among many that increases the risk of bone fracture over time [1]. Bone mineral density (BMD) may be measured using a dual-energy X-ray absorptiometry (DEXA) scan. Interpretation of DEXA scan results among postmenopausal women compares BMD measurements to the mean bone density of a young, healthy population: a normal BMD is defined as greater than -1 standard deviation (SD), osteopenia as -1 to -2.4 SD, and osteoporosis as ≤-2.5 SD from the reference standard. The prevalence of osteoporosis among women aged 65 and older is 27.1%. Osteoporosis is associated with increased risk of fragility fractures, defined as bone fractures that occur spontaneously or as a result of less force than would typically cause a fracture, such as a fall from a standing position. Consequently, the US Preventive Services Task Force recommends routine screening for osteoporosis in women aged 65 and older and in postmenopausal women with one or more risk factors for osteoporosis (Grade B recommendation) [2].

However, a subgroup of women with bone density in the osteopenic range is also at significantly increased risk of a fragility fracture. These women may be identified by one of several validated instruments, including the widely used Fracture Risk Assessment Tool (FRAX) score. The FRAX calculator estimates the 10-year risk of a major osteoporotic fracture and of a hip fracture. It was originally created to allow for fragility fracture risk assessment both with and without bone densitometry to support lower resource regions, but it is more accurate when BMD is included. It has an area under the curve (AUC) of 0.65-0.67 for major osteoporotic fractures (MOF) and 0.76 to 0.79 for hip fractures. Commonly accepted risk thresholds for treatment to prevent MOF and hip fractures are 20% and 3%, respectively [2,3]. A registry-based cohort study found the overall prevalence of a FRAX-predicted MOF score ≥20% to be 21.4% for women aged 80 years and older. Compared to women aged 60-69 years old, the number-needed-to-screen for an MOF risk ≥20% among women 80 years and older was two versus thirty, with an accuracy of 58.6% and 87.8%, respectively [4].

## Case presentation

A 92-year-old female with a history of hyperlipidemia, hypothyroidism, impaired glucose tolerance, disequilibrium, hypovitaminosis D, and osteopenia was seen for a routine annual visit. Her medications included atorvastatin, levothyroxine, aspirin, a multivitamin, and a supplement containing 10 mcg (400 IU) of vitamin D3 and 600 mg of calcium carbonate. Her past medical history was negative for previous fracture, parental hip fracture, extended treatment with glucocorticoids, tobacco use, rheumatoid arthritis, secondary causes of bone loss, and regular alcohol intake. She lived at home and had good support systems, including regular assistance with activities of daily living (ADLs). Her most recent vitamin D, thyroid-stimulating hormone, and parathyroid hormone levels were within normal limits (Table 1).

**Table 1:** Lab values prior to fragility fracture

Test	Result	Normal range
Vitamin D	41 ng/mL	30-100 ng/mL
Thyroid stimulating hormone	4.28 mUI/L	0.4-4.50 mIU/L
Parathyroid hormone	28 pg/mL	11 - 51 pg/mL

There was no record of hypercalcemia over several years. A DEXA scan performed two and a half years previously showed a T-score of 0.3 SD for the total hip, a T-score of 1.3 SD at the lumbar spine, and a T-score of -1.1 SD at the left femoral neck. Using the FRAX score, her 10-year risk of a MOF was 9.8%, and her hip fracture risk was 2.6%. These results represented a two percent increase in bone density at the lumbar spine in comparison to a DEXA scan performed five years earlier with similar equipment (Hologic, Inc., MA, USA). Agreement to continue screening for osteoporosis was achieved through shared decision-making, including a discussion of the limited data available regarding the effectiveness of treatment to prevent fragility fractures in people over 90 years of age.

Three days later, new DEXA scan results showed a bone density at the left femoral neck in the osteopenic range (0.735 g/sq cm, T-score =-1.0) and within normal limits at the lumbar spine (1.262 g/sq cm, T-score=2.0). Within a week of this study, the patient experienced severe back pain rated eight to nine out of 10 without a precipitating event.

**Figure 1:**
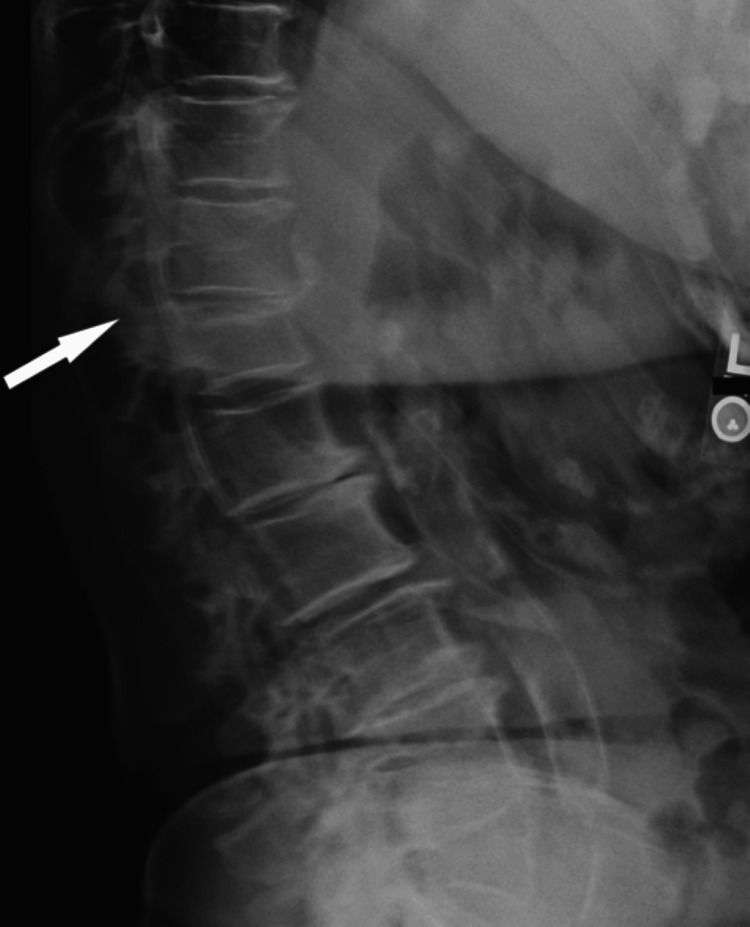
X-ray showing an acute upper endplate vertebral compression fracture at T12 (arrow)

The following day, a lumbar X-ray showed disk space narrowing at multiple levels, spondylolisthesis at L4/5, diffuse spondylosis, and a new compression fracture at the upper endplate of T12 (Figure 1). Subsequently, she endured a prolonged course of ongoing back pain that was treated with multiple modalities, including a brief course of narcotics, physical therapy, a back brace, and nasal calcitonin, but she had no other direct sequelae. She continues to see a pain management specialist for back pain and receives denosumab for secondary prevention of fragility fractures.

## Discussion

This case describes a patient with a reportedly normal lumbar spine bone density on a DEXA scan who experienced a spontaneous fragility fracture soon thereafter.

Degenerative changes of the lumbar spine may reduce the accuracy of BMD measurements, giving false reassurance about the magnitude of fragility fracture risk [5]. In this case, the DEXA scan results showed osteopenia at the hip and a deceptively normal bone density result at the lumbar spine for the patient’s age. A prospective study of 1,044 women over 75 years and older followed for 10 years found the progression of degenerative disease of the lumbar spine to be associated with increased BMD and a lower incidence of osteoporosis in comparison to women without degenerative changes of the lumbar spine at baseline [6]. Osteoarthritic changes are the most common cause of artifacts on DXA assessment, especially in elderly patients [7]. Aortic calcifications have a minimal influence on BMD in the majority of cases but may increase BMD by 5%. However, the effect of this artifact is considered minimal and rare [8]. In summary, a discrepancy between spine and femoral neck bone density assessments should raise concern of erroneous spinal measurement affected by degenerative change, especially in the elderly. In these patients, bone density at the femoral neck may be more reliable for predicting fracture risk and monitoring response to treatment. The prevention of fragility fractures is a significant health concern: it is estimated that 40-60% of people who suffer a hip fracture lose some ability to perform ADLs [2]. Vertebral fractures, both clinical and subclinical, confer a five-fold increase in subsequent vertebral fractures and a two- to threefold increase in MOF [3]. Approximately 30% of MOF and 60% of hip fractures occur in women 80 years and older [9]. Anti-resorptive agents alendronate, risedronate, and zoledronic acid reduce hip fractures by approximately 40% and all non-vertebral fractures by 20-30% among post-menopausal women [1]. Data from clinical trials with fracture endpoints, including women aged 90 years and older, the frail elderly, and those living in residential facilities, are limited.

The Fracture Intervention Trial, the pivotal clinical trial for alendronate, which is the most prescribed oral antiresorptive agent, enrolled post-menopausal women < 80 years of age. Studies of other treatment alternatives included subpopulations of women 75 or 80 years of age and older, typically with either a T-score of -2.5 at the femoral neck with or without an additional risk factor for fragility fractures and/or a history of fragility fracture. Most studies were three years in duration.

A randomized, placebo-controlled trial (RCT) of the bisphosphonate risedronate in women ≥ 80 years old showed a non-statistically significant reduction (risk reduction (RR) 0.8; 95% confidence interval (CI) 0.6-1.2, p=0.35) in hip fractures. In a post-hoc pooled analysis of three placebo-controlled RCTs, treatment with risedronate significantly reduced the incidence of vertebral fracture (harm reduction (HR)=0.56, 95% CI 0.39-0.81, p=0.003). Similarly, an RCT with zoledronic acid, a parenteral bisphosphonate, in women ≥ 75 years also found a significant reduction in vertebral fractures (HR 0.34, 95% CI 0.21-0.55, p< 0.001) and a non-significant reduction in hip fractures (HR=0.82, 95% CI 0.56-1.2, p=0.297) [9]. Updated meta-analyses confirm that bisphosphonates such as alendronate, risedronate, and zoledronic acid significantly reduce secondary fracture risk, including a marked decrease in vertebral and non-vertebral fractures across diverse patient populations [10].

Denosumab, a parenteral inhibitor of RANK/RANK ligand, in women aged ≥ 75 years found a significant reduction in vertebral fractures in an RCT (RR=0.36, 95% CI 0.25-0.53). A post-hoc analysis of the same study showed a significant reduction in the incidence of hip fractures as well (denosumab 0.9%, placebo 2.3% absolute risk reduction (ARR)=1.4%, p< 0.01 [9]. Additionally, pooled data from multiple randomized trials reinforce the robust anti-fracture efficacy of denosumab, with significant reductions in vertebral, hip, and non-vertebral fractures sustained over long-term treatment [11,12].

An RCT of teriparatide, a biosynthetic parathyroid hormone analog, in women aged ≥ 75 years over 19 months, showed a significant reduction in vertebral fractures (RR=0.35, p< 0.05) [9]. Teriparatide continues to demonstrate benefit in fracture healing and prevention; recent studies in elderly populations report accelerated healing times and improved functional recovery in addition to lowering vertebral fracture incidence [13].

Recent RCTs have also highlighted the role of newer anabolic agents such as romosozumab, which has been shown to both stimulate bone formation and inhibit bone resorption, leading to significant reductions in vertebral and clinical fractures among postmenopausal women at high risk. Sequential therapy starting with romosozumab followed by alendronate has demonstrated superior fracture prevention compared to alendronate alone [14].

Data on the efficacy of primary and secondary prevention of fragility fractures and other outcomes among the very elderly are evolving. A recent retrospective study presented at the ENDO 2025 symposium in July compared 44,338 patients treated with bisphosphonates, denosumab, raloxifene, or teriparatide paired with propensity-score matched controls. Those treated experienced fewer hospitalizations (odds ratio (OR) 0.81, 95% CI 0.79-0.84) and risk of death (OR 0.85, 95% CI 0.83-0.88) over five years [15].

As for this patient, two and a half years after her initial presentation with a fragility fracture of the lumbar spine, she has generally maintained her prior level of function and finds her back pain to be manageable.

## Conclusions

The risk assessment for fragility fracture in a 92-year-old patient was confounded by degenerative changes in the spine. Such changes can falsely increase BMD measurements. In this instance, a spontaneous vertebral fracture followed a normal vertebral bone density finding by several days. More research is needed to improve both risk assessment and fragility fracture prevention in the very elderly.

## References

[1] Reid IR, Billington EO (2022). Drug therapy for osteoporosis in older adults. Lancet.

[2] Nicholson WK, Silverstein M, Wong JB (2025). Screening for osteoporosis to prevent fractures: US Preventive Services Task Force recommendation statement. JAMA.

[3] LeBoff MS, Greenspan SL, Insogna KL, Lewiecki EM, Saag KG, Singer AJ, Siris ES (2022). The clinician's guide to prevention and treatment of osteoporosis. Osteoporos Int.

[4] Crandall CJ, Schousboe JT, Morin SN, Lix LM, Leslie W (2019). Performance of FRAX and FRAX-based treatment thresholds in women aged 40 years and older: the Manitoba BMD registry. J Bone Miner Res.

[5] Schneider DL, Bettencourt R, Barrett-Connor E (2006). Clinical utility of spine bone density in elderly women. J Clin Densitom.

[6] Tenne M, McGuigan F, Besjakov J, Gerdhem P, Åkesson K (2013). Degenerative changes at the lumbar spine--implications for bone mineral density measurement in elderly women. Osteoporos Int.

[7] Stewart A, Black AJ (2000). Bone mineral density in osteoarthritis. Curr Opin Rheumatol.

[8] Cherney DD, Laymon MS, McNitt A, Yuly S (2002). A study on the influence of calcified intervertebral disk and aorta in determining bone mineral density. J Clin Densitom.

[9] Vandenbroucke A, Luyten FP, Flamaing J, Gielen E (2017). Pharmacological treatment of osteoporosis in the oldest old. Clin Interv Aging.

[10] Shi L, Min N, Wang F, Xue QY (2019). Bisphosphonates for secondary prevention of osteoporotic fractures: a Bayesian network meta-analysis of randomized controlled trials. Biomed Res Int.

[11] Cummings SR, San Martin J, McClung MR (2009). Denosumab for prevention of fractures in postmenopausal women with osteoporosis. N Engl J Med.

[12] Boonen S, Adachi JD, Man Z (2011). Treatment with denosumab reduces the incidence of new vertebral and hip fractures in postmenopausal women at high risk. J Clin Endocrinol Metab.

[13] Kim SJ, Park HS, Lee DW, Lee JW (2019). Short-term daily teriparatide improve postoperative functional outcome and fracture healing in unstable intertrochanteric fractures. Injury.

[14] Saag KG, Petersen J, Brandi ML (2017). Romosozumab or alendronate for fracture prevention in women with osteoporosis. N Engl J Med.

[15] Flocco G Pharmacological Treatment Appears to Benefit Patients 80 or Older With Fracture. https://www.healio.com/news/endocrinology/20250713/pharmacological-treatment-appears-to-benefit-patients-80-or-older-with-fracture#:~:text=“Patients%20with%20osteoporosis%20who%20were,Flocco%20said%20during%20the%20presentation%20accessed%208/10/25..

